# A Narrative Review of the Roles of Chondromodulin-I (Cnmd) in Adult Cartilage Tissue

**DOI:** 10.3390/ijms25115839

**Published:** 2024-05-27

**Authors:** Viviana Reyes Alcaraz, Girish Pattappa, Shigenori Miura, Peter Angele, Torsten Blunk, Maximilian Rudert, Yuji Hiraki, Chisa Shukunami, Denitsa Docheva

**Affiliations:** 1 Department of Musculoskeletal Tissue Regeneration, Orthopaedic Hospital König-Ludwig-Haus, University of Würzburg, 97070 Würzburg, Germany; tania.reyes-alcaraz@uni-wuerzburg.de (V.R.A.); girish.pattappa@uni-wuerzburg.de (G.P.); 2 Department of Molecular Biology and Biochemistry, Division of Dental Sciences, Graduate School of Biomedical and Health Sciences, Hiroshima University, Hiroshima 734-8553, Japan; miuras@hiroshima-u.ac.jp (S.M.); shukunam@hiroshima-u.ac.jp (C.S.); 3 Laboratory of Experimental Trauma Surgery, Department of Trauma Surgery, University Hospital Regensburg, 93053 Regensburg, Germany; angele@sporthopaedicum.de; 4 Department of Trauma, Hand, Plastic and Reconstructive Surgery, University Hospital Würzburg, 97080 Würzburg, Germany; blunk_t@ukw.de; 5 Department of Orthopaedics, Orthopaedic Hospital König-Ludwig-Haus, University of Würzburg, 97070 Würzburg, Germany; m-rudert.klh@uni-wuerzburg.de; 6 Department of Cellular Differentiation, Institute for Frontier Medical Sciences, Kyoto University, Kyoto 606-8501, Japan; hiraki.yuji.32m@st.kyoto-u.ac.jp

**Keywords:** chondromodulin, articular cartilage, osteoarthritis

## Abstract

Articular cartilage is crucial for joint function but its avascularity limits intrinsic repair, leading to conditions like osteoarthritis (OA). Chondromodulin-I (Cnmd) has emerged as a key molecule in cartilage biology, with potential implications for OA therapy. Cnmd is primarily expressed in cartilage and plays an important role in chondrocyte proliferation, cartilage homeostasis, and the blocking of angiogenesis. In vivo and in vitro studies on Cnmd, also suggest an involvement in bone repair and in delaying OA progression. Its downregulation correlates with OA severity, indicating its potential as a therapeutic target. Further research is needed to fully understand the mode of action of Cnmd and its beneficial implications for managing OA. This comprehensive review aims to elucidate the molecular characteristics of Cnmd, from its expression pattern, role in cartilage maintenance, callus formation during bone repair and association with OA.

## 1. Introduction

**Figure 1:**
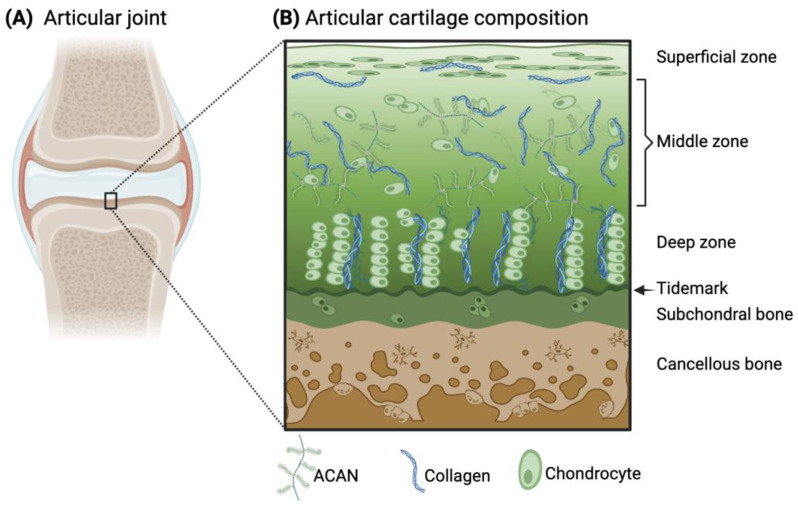
Schematic illustration of the articular joint (**A**) and the microscopic structure and composition of the articular cartilage and subchondral bone (**B**). ACAN and collagen are the main components of the cartilage ECM, arranged differently throughout the zones of articular cartilage. The principal resident cells in cartilage are chondrocytes, whose morphology also varies within the tissue. (**B**) Adapted from [3] and licensed under creative-common-license CC BY 4.0.

Articular cartilage is an avascular and aneural connective tissue that covers synovial joints to enable friction-free joint movement and prevent high stresses from transferring to the underlying subchondral bone. The extracellular matrix (ECM) of articular cartilage primarily consists of collagens (predominantly type II (Col2)), elastin fibers, glycoaminoglycans (e.g., chondroitin sulfate), and proteoglycans (e.g., aggrecan (ACAN)). The cell population within the tissue that deposits and maintains the ECM is mainly composed of chondrocytes. Within the tissue, there is an intrinsic anisotropy of the cartilage structure with depth and in the surrounding proximity of the chondrocytes, which provides cartilage with its unique mechanical properties. Specifically, the presence of negatively charged proteoglycans attracts water molecules into the tissue during joint movement, while towards the subchondral bone interface, there are greater amounts of proteoglycans and thicker collagen fibrils (Figure 1). Due to matrix restrictions, the water concentration decreases with depth, which leads to the creation of a swelling pressure that helps withstand high compressive loads during joint movement [1,2]. Furthermore, cartilage is critical to the formation of the long bones, serving as a template for the process of endochondral ossification.

However, the avascularity of cartilage limits its capacity for intrinsic repair and makes it susceptible to further degeneration upon damage (e.g., trauma or sports injury). Moreover, the natural repair mechanism tends to result in fibrocartilage formation, further exacerbating disease pathology. One of the major pathological conditions associated with articular cartilage is osteoarthritis (OA) [4]. OA is a degenerative joint disease characterized by the breakdown and loss of cartilage over time, leading to joint pain, stiffness, and impaired joint function. In order to develop innovative therapies, understanding the complex nature of articular cartilage and the pathogenesis of OA would help to improve the quality of life for individuals affected by this disease.

Avascular cartilage expresses the molecule chondromodulin-I (Cnmd) [5,6]. It was first discovered as a 25 kDa glycoprotein by Hiraki et al. and is predominantly found in cartilage, from which its name is derived [7]. Since its discovery, Cnmd has been investigated in terms of gene locus, protein structure, tissue and cell expression. In terms of its function, Cnmd has been proven to synergistically promote proliferation of cultured chondrocytes in the presence of *FGF*-2 [7,8], play an active role in cartilage homeostasis [9], and inhibit angiogenesis [10,11]. However, despite these discoveries, there are several open questions, for example, protein domain structure and direct binding partners, Cnmd-dependent signaling pathways, as well as its impact on OA progression or amelioration using adequate OA models in vivo.

This review presents a concise exploration of Cnmd-related studies, a promising yet mysterious cartilage-specific marker. The main focus was to summarize the current understanding of Cnmd, and the review was structured as follows: a brief introduction on articular cartilage and OA pathogenesis; review scope and research strategy (Section 1 and Section 2); existing knowledge on Cnmd molecular characteristics and expression patterns (Section 3); Cnmd and its critical role in articular cartilage maintenance and bone repair (Section 4); link between Cnmd and OA (Section 5); and conclusion and outlook (Section 6).

**Figure 2:**
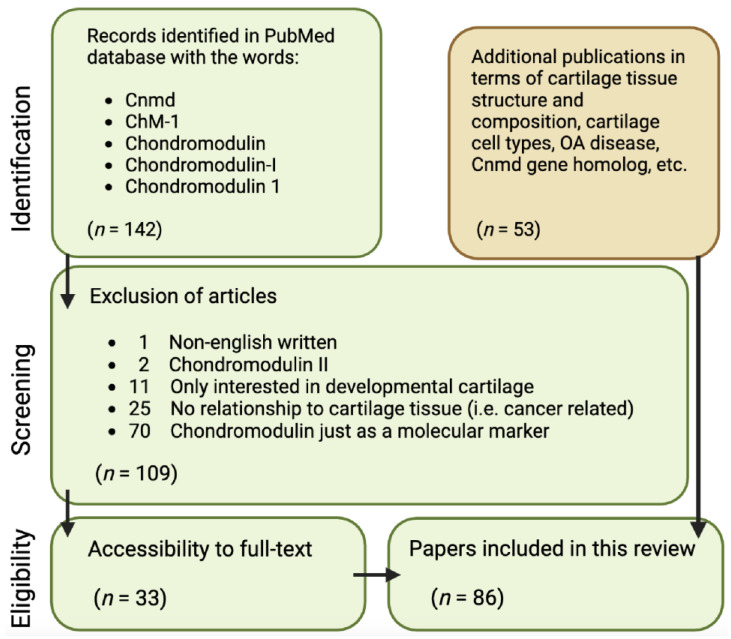
Flowchart of article identification and selection strategy for publications used in this concise review.

It is aimed at critically discussing areas for further research on Cnmd as well as highlighting Cnmd’s importance in cartilage biology and its potential role in mitigating OA. To conduct this analysis, the following approach was employed, examining all publications available in the PubMed database starting in November 2022: using the keywords “Chondromodulin”, “Chondromodulin-1”, “Chondromodulin-I”, “ChM-1”, and “Cnmd”, a total of 142 papers were retrieved. Through a refinement process outlined in Figure 2, 109 papers were excluded due to being written in a non-English language; Cnmd solely as a molecular marker (mainly via RT-PCR validation of expression); being related to chondromodulin-II; or lacking relevance to cartilage tissue. This resulted in 33 Cnmd-related papers being the basis of this review. Lastly, additional 53 publications were cited in terms of cartilage tissue structure and composition, cartilage cell types, OA disease, and the *Cnmd* homolog gene.

## 2. Knee Articular Cartilage

### 2.1. Structure and Composition

Articular cartilage is a form of hyaline cartilage with a thickness ranging from 2–4 mm. Unlike other musculoskeletal tissues, such as bone or skin, there are few blood vessels and nerves within the articular cartilage structure. The ECM of articular cartilage is primarily composed of water, collagens (II, III, VI, XII, XIV, and XVI), and proteoglycans (mainly ACAN). Collagen fibrils represent more than 50 percent of the non-wet constituents, and Col2 is by far the most abundant in articular cartilage. Col2 is a homotrimer formed by three identical alpha1(II) chains; it acts as an anchoring site for chondrocytes and growth factors to attach in the ECM. This component not only participates as a structural element but it has also been considered an important signaling molecule for chondrocytes [12]. Another major component is ACAN. This large aggregated proteoglycan is an essential binding site for water molecules to be trapped within the cartilage matrix [13]. Thus, water, Col2, and ACAN constitute the main elements that provide the structural integrity of the tissue and its distinct viscoelastic properties [14].

Additionally, the ECM contains in smaller quantities other non-aggregating proteoglycans (e.g., decorin, biglycan, and fibromodulin) and non-collagenous proteins (e.g., fibronectin, matrillin-3, cartilage oligomatrix protein (COMP)) that also contribute to the overall function and integrity of the tissue [15,16].

The primary components of articular cartilage (i.e., Col2, ACAN, and chondrocytes) are arranged in a specific way that varies with the depth of the tissue and play a critical role in maintaining its functional properties. Specifically, to withstand mechanical stress and provide a smooth surface for joint movement. The specific zones of cartilage are the superficial, middle, deep, and calcified zones (Figure 1). Starting from the superficial zone, which represents only 10% of the total thickness, there are densely layered and packed collagen fibers aligned parallel to the cartilage surface with minimal proteoglycan content [1]. One essential glycoprotein within the superficial zone is lubricin, which provides lubrication of the cartilage surface during loading [17]. This surface region is the layer that interacts with molecules present in the synovial fluid. Below this region, the middle zone (or transitional zone) covers about 50% of the total tissue, and it contains randomly distributed collagen fibers and proteoglycans. Next, the deep zone represents approximately 30–40% of the total thickness of articular cartilage. Within this zone, chondrocytes are grouped in pillars or columns perpendicular to the articular surface, together with thick collagen fibrils that are vertically arranged. Interspersed between the chondrocytes and collagen fibers, the proteoglycans are randomly distributed in this region and are at their highest concentration within the whole cartilaginous tissue [18,19,20]. In close contact with the subchondral bone, the calcified zone contains rounded chondrocytes and collagen fibers anchored perpendicularly to the calcified surface and a low concentration of proteoglycans [1]. All together, this anisotropic structure enables articular cartilage to withstand both compressive and shear forces during joint motion.

### 2.2. Cell Types and Key Molecules

Chondrocytes are the primary cell population within articular cartilage and are the progeny of mesenchymal stem cells (MSCs) derived from the embryonic mesoderm or neural crest. These are ovoid cells with a maximum diameter in vivo of about 10 µm [21]. The articular cartilage structure creates a low-oxygen environment for chondrocytes, ranging from 4% in the superficial layer to 1% in the deepest region [3]. The chondrocytes play a role in the structural formation of different regions of articular cartilage, with their morphology and function depending upon their location. The superficial layer contains flattened chondrocytes, whereas the chondrocytes in the middle zone are more spherical. In the deep zone, the chondrocytes are organized in columns that run parallel to the collagen fibers but perpendicular to the joint line. In contrast, the calcified zone is sparsely populated with larger hypertrophic chondrocytes [20]. 

Besides the presence of mature chondrocytes in every region of articular cartilage, recent studies have described the presence of cartilage progenitor cells (CPCs), both in healthy and diseased cartilage, as a subpopulation with stem cell-like characteristics such as migratory activity, clonogenic abilities, and multipotency [22,23,24]. Recent investigations have identified this sub-population to be present in the superficial layer of the tissue and found that they produce a more stable articular cartilage compared to that derived from bone marrow mesenchymal stromal cells, which are predisposed towards hypertrophic chondrocyte differentiation [24,25]. In a pathologic context (e.g., OA), CPCs seem to migrate from the vessels of the subchondral bone, expressing the chondrogenic Sex-determining Region Y (SRY)-Box9 (SOX9) and runt-related transcription factor 2 (RUNX2), associated with osteoblast. However, the effect of CPCs on regeneration mechanisms is scarce due to the fact that CPCs mainly produce type I collagen (COL1) in vivo [25,26].

Signaling factors are the protagonists in modulating cell–cell signaling and cell-matrix interactions during the development and maturation of articular cartilage. There are a great number of growth and transcription factors that are known to be involved in chondrogenesis and cartilage homeostasis [27]. In short, chondrogenesis involves the condensation of MSCs coming from the lateral plate mesoderm to be differentiated into rounded chondrocytes and produce the specific ECM. Growth factors ranging from Transforming Growth Factor-β (e.g., TGFβ-1), Fibroblast Growth Factor (e.g., FGF-2), Bone Morphogenic Proteins (BMPs), and Insulin Growth Factors (IGFs) are involved. During the condensation process, the transcription factor SOX9 is expressed alongside SOX5/-6 to initiate the chondrogenic differentiation process [28]. Due to its role in early cartilage development, SOX9 is strongly related to the upregulation of ECM components like COL2a1 and ACAN [29]. These genes have been used as markers for in vitro chondrogenic assays to show that cells are differentiating towards the chondrogenic lineage. Additionally, during the condensation and differentiation phases, processes are also mediated by the ECM with receptors that initiate downstream signaling pathways [30,31]. 

A molecular factor that has been shown to be expressed during early cartilage formation is Cnmd. It has been demonstrated that Cnmd can prevent angiogenesis by inhibiting angiogenic factors (e.g., vascular endothelial growth factor (VEGF)) as well as chondrocyte hypertrophy [32,33]. Interestingly, the in vivo analyses of Cnmd knockout (KO) mice postulated that Cnmd is a dispensable factor in cartilage development [34,35,36]. These studies have shown that Cnmd KO mice are viable and fertile with a normal life span and show no profound developmental phenotypes, namely no obvious changes in endochondral bone formation, cartilage deformations during development, or abnormal vascular invasion. 

### 2.3. Pathologic Changes in Osteoarthritis

OA is a complex and chronic disease that affects all of the joint tissues within the knee and not solely the articular cartilage [4]. According to the World Health Organization (WHO), approximately 528 million people around the globe suffered from some level of OA in 2019 [37]. This disease is influenced by a variety of parameters, including age, gender, genetic predisposition, mechanical factors (e.g., body mass index (BMI), joint malalignment), or previous traumatic joint injuries. 

In healthy cartilage, chondrocytes are in a relatively “resting” state, and their ECM undergoes minimal turnover. However, OA induces a notable shift towards “active” chondrocytes, whereby there is a shift in their metabolic activity, leading to significant matrix remodeling via stimulation of catabolic factors (e.g., MMP-13) that further leads to cartilage calcification [4]. The breakdown of the cartilage structure is thus attributable to the increased production of matrix-degradative enzymes and the reduced matrix synthesis by chondrocytes. The cartilage gradually loses its ability to withstand mechanical stress and to provide adequate cushioning, leading to joint pain, stiffness, and functional impairment. 

**Figure 3:**
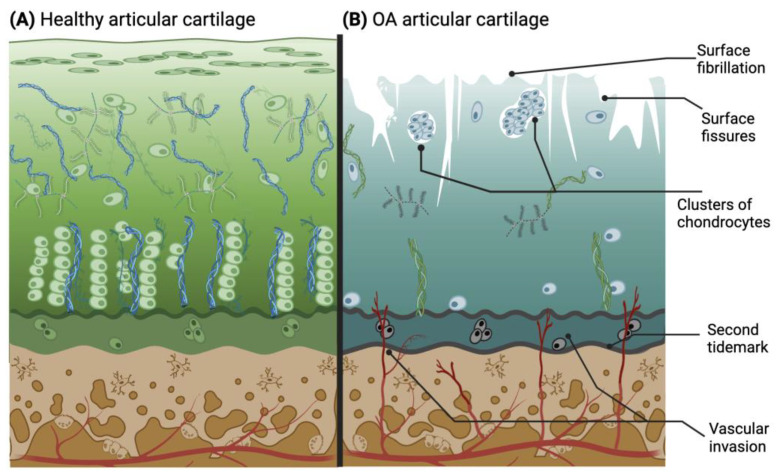
Drawing of histological characteristics in healthy (**A**) and osteoarthritic (**B**) articular cartilage. The latter illustrates the main pathological changes in cartilage during OA progression. Adapted from [3,42] and licensed under creative-common-license CC BY 4.0.

The rate of knee OA progression is relatively slow and can potentially take between 10 and 15 years to fully develop [38]. During this time, the stages can be categorized as early, moderate, and late OA, based on the visible characteristics of the joint. Early OA may be the most difficult stage to diagnose due to the sporadicity of symptoms [39]. Nevertheless, some clinical criteria help to identify this early stage, since it is typically found in patients over 50 years old and can be detected through physical examination, endoscopy, and revision of the patient’s history. Some of the criteria are stiffness no longer than 30 min, pain, and joint crackling sounds (crepitus) [40]. In the early stages of OA, clinical diagnostics play a central role due to the ongoing exploration of biological markers. A potential biological marker for early OA is interleukin-1β (IL-1β) [41]. In the moderate and late OA stages (Figure 3B), the microscopic changes involve deterioration or fibrillations of the superficial zone of articular cartilage that extend into the transition zone. Additionally, histological stainings demonstrate a significant reduction in proteoglycan content in both the superficial and transitional zones. Another characteristic found in late OA is the duplication of the tidemark that establishes the limit between the calcified zone and the non-calcified zone [42,43,44]. Furthermore, blood vessels from the subchondral bone penetrate through the tidemark, and there are structural alterations in the subchondral bone. As the condition advances, the surface irregularities grow larger, and a greater proportion of the superficial layer becomes eroded away. The fibrillations extend deeper into the cartilage, eventually leading to fissures that reach the subchondral bone [18]. This pathological microenvironment disrupts the metabolic activity of chondrocytes, resulting in mitochondrial dysfunction that initiates an increase in reactive oxygen species (ROS), subsequently inducing chondroptosis and further depletion of cells in the hypertrophic layer [45]. Thus, the natural chondrocyte columnar organization created in the tissue is irreversibly lost with degeneration.

Understanding the dynamic changes that occur in articular cartilage during the progression of OA is essential for developing targeted therapies. Genetic factors and specific inflammatory cytokines (e.g., IL-1β, TNF-α) play major roles in OA pathogenesis, influencing an individual’s susceptibility, the regulation of chondrocyte activity and ECM remodeling [46]. The changes in the environment lead to alterations in chondrocyte metabolism. Specifically, chondrocytes begin to undergo a process of senescence, whereby chondrocytes change their morphology and adopt a senescence-associated secretory phenotype (SASP), leading to the release of pro-inflammatory cytokines (e.g., IL-1β and TNF-α), chemokines (e.g., MCP-1), and matrix degradative enzymes (e.g., MMP-13 and ADAMTS-5) [47,48,49]. These senescent chondrocytes remain within the tissue due to stimulation of pro-survival pathways (e.g., PI3K) that inhibit apoptotic genes (e.g., caspase-3) and prevent their death [50]. However, with the advancement of the OA process, tissue degeneration continues, and gradually, chondrocytes enter apoptosis.

Targeting the processes that alter chondrocyte metabolic activity has the potential to restore the balance between matrix synthesis and degradation, thus slowing disease progression, whilst promoting cartilage repair and alleviating symptoms. Current pharmacological therapies are being developed to help reduce the symptoms of OA, either via prevention of cytokine presence (e.g., anti-TNF), senolytic drugs (e.g., UBX0101) that remove senescent cells, or enzymatic inhibitors (e.g., ADAMTS inhibitors). These drugs have shown promise in clinical trials, although they have yet to reach the commercial market [51,52]. In this context, no studies have focused on Cnmd for therapeutic use, and thus, this review paper wishes to explore its therapeutic potential and address its importance with respect to post-natal cartilage homeostasis.

## 3. Cnmd: Gene and Protein

### 3.1. Gene: Discovery and Nomenclature

Chondromodulin-I (now Cnmd but formerly known as ChM-1, Leukocyte cell-derived chemotaxin-I (LECT1), and BRICD3) was found and cloned for the very first time by Hiraki et al. (1991). Hiraki’s group purified CNMD from fetal bovine epiphyseal cartilage and recognized Cnmd as a tissue-specific functional matrix component [7].

**Figure 4:**
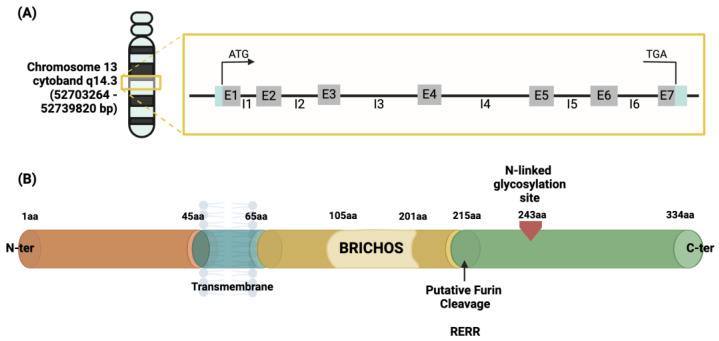
Schematic representation of the human *CNMD* gene (**A**) and its precursor protein (**B**). The gray boxes containing the letter ‘E’ in (**A**) represent one of the seven exons constituting the gene, divided by the introns represented by the letter ‘I’. Once translated, the precursor is a type II glycoprotein consisting of 334 amino acids (**B**), which begins from the N-terminal with a transmembrane region crossing from amino acid 45 to 65, followed by a BRICHOS domain ranging from amino acid 105 to 210, and an N-glycosylated site in amino acid 243 from which the mature form of CNMD originates. This domain is released by protease cleavage, namely the intracellular furin, using the RERR recognition site in position 215 of the amino acid sequence of the CNMD precursor. Abbreviations: bp (base pair); aa (amino acid).

The human *CNMD* gene has been located on chromosome 13, more specifically within the cytogenetic band 14.3, chromosome location 52703264–52739820 bp [53,54]. It consists of seven exons and accounts for approximately 40 kb, containing a TATA-less type promoter. Exons 6 and 7 encode for the C-terminal domain of the CNMD precursor protein [55,56]. The description is represented in Figure 4A. This gene encodes a protein containing 334 amino acids. This is identified as the origin of the mature form of the CNMD protein.

### 3.2. Cnmd Precursor and Mature Protein

**Figure 5:**
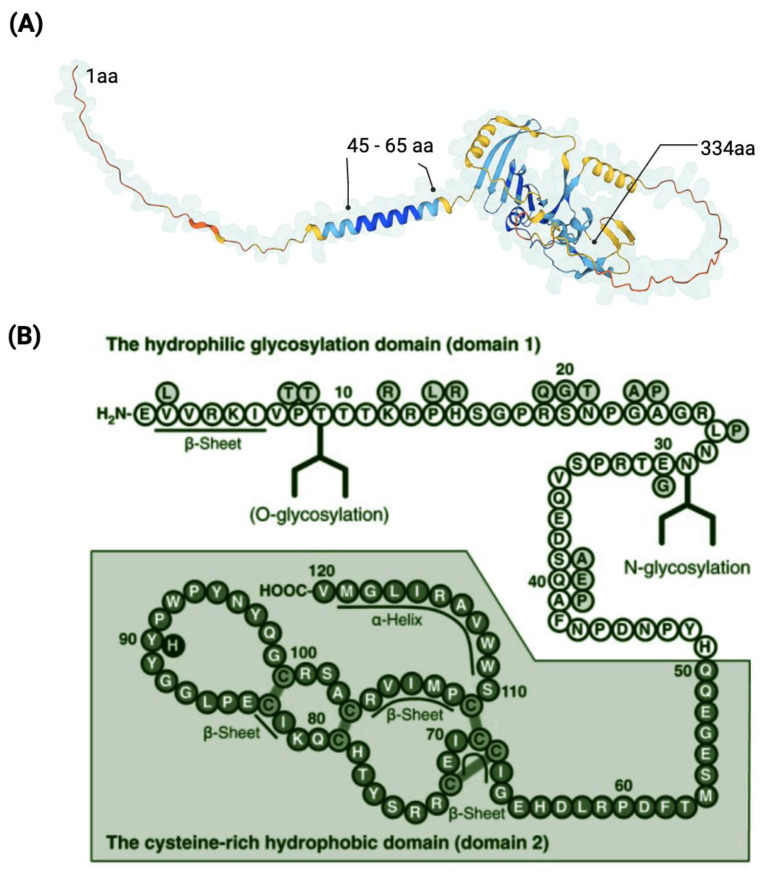
Representation of the CNMD protein. Three-dimensional model of the precursor CNMD. It indicates the starting point (1 aa), the transmembrane region (45–65 aa), and the ending point (334 aa) of CNMD. Source: https://alphafold.ebi.ac.uk/entry/O75829 (accessed on 11 February 2023) (**A**). Amino acid sequence of human mature CNMD (**B**). The mature form of Cnmd contains 120 amino acids and consists of the hydrophilic domain 1 and the cysteine-rich hydrophobic domain 2. (**B**) Adapted from [64] and licensed under creative-common-license CC BY 2.5 DEED/cropped from original and color-modified.

The initial 334 amino acid (aa) sequence is a type II transmembrane glycoprotein (Figure 4B). Previous protein sequence analyses suggest the following structural features for the human precursor CNMD protein: starting from the N-terminus, a transmembrane region is positioned between amino acids 45–65. Prior to the cleavage site, a BRICHOS domain is included from amino acid residues 104 to 201 [57]. The BRICHOS domain has been found via homology search in the data banks UniProtKB and GenomeLKPG in eight proteins, but its function has not been experimentally explored. Intriguingly, the unrelated BRICHOS-containing protein families have been associated with a variety of diseases, such as dementia, cancer, and respiratory distress [58,59]. From position 215, which is the furin cleavage site, the mature form, the C-terminal domain, of the CNMD protein begins, and the N-glycosylation site is located [33]. Theoretical models have predicted the three-dimensional conformation of CNMD. In particular, Figure 5A depicts the structure generated by the AlphaFold website [60,61]. This model reveals the following distinct structural features: alpha-helices (regions 45–65 aa and 110–120 aa) and three beta-sheets from 70–100 aa. Eight highly conserved cysteine residues contribute to four disulfide bridges in the C-terminal domain, thus leading to a globular and stable form of the mature CNMD protein.

CNMD shares high homology with one gene, tenomodulin (Tnmd), and this novel protein family of two members is only found in vertebrates. Overall, Cnmd and Tnmd are 33% identical [62,63], while their C-terminal cysteine-rich domains are 77% similar. In contrast to Cnmd, Tnmd is a tendon-specific protein. It has not been fully clarified whether they have overlapping expression sites and engage in compensatory mechanisms.

With regards to alternative splicing and the existence of isoforms, there are so far no articles reporting such for Cnmd protein.

The mature form of human CNMD is encoded by the C-terminal domain of its larger precursor [7]. Human mature CNMD (and its equivalents in other animal species) exists as a single chain of 120 amino acids with a molecular mass of approximately 25 kDa. The molecular structure of the mature CNMD consists of two principal domains (Figure 5B): the hydrophilic glycosylation domain (domain 1), made of 50 amino acids, is the hydrophilic region that encloses the N-glycosylated site, and the cysteine-rich hydrophobic domain (domain 2) (the remaining 70 amino acids) comprises the C-terminal of the protein, which is a hydrophobic region with four disulfide bonds, essential for the anti-angiogenic action of mature Cnmd [5,55,64]. Eight cysteine residues are well conserved across species [65]. 

The processing of the Cnmd precursor occurs intracellularly, and once cleaved at the predicted furin site, the C-terminal of the larger precursor converts into the mature Cnmd [66]. This is the 25 kDa glycoprotein that has been found in the ECM of avascular cartilage and has been shown to stimulate chondrocyte proliferation and maturation but inhibit angiogenesis [67]. However, Cnmd is sensitive to additional cleavage of the mature form that results in a 14 kDa inactive form of Cnmd found in the calcified zone of cartilage [5].

Despite the knowledge gained on Cnmd proteins, there are several topics to be resolved in the field, i.e., (i) the three-dimensional conformation of Cnmd remains speculative, and performing crystallographic analysis would be very necessary; (ii) the intracellular Cnmd localization and deposition path outside of the cells need to be clarified; (iii) the factors leading to the inactivation, by secondary cleavage, of the mature Cnmd are to be discovered; (iv) proteins that co-localize with Cnmd in the ECM are also to be identified; and (v) Cnmd-dependent protein-to-protein interactions should also be addressed. In sum, structural biology techniques and targeted functional assays are necessary to further elucidate the above aspects of Cnmd protein.

### 3.3. Cnmd Expression Pattern

**Figure 6:**
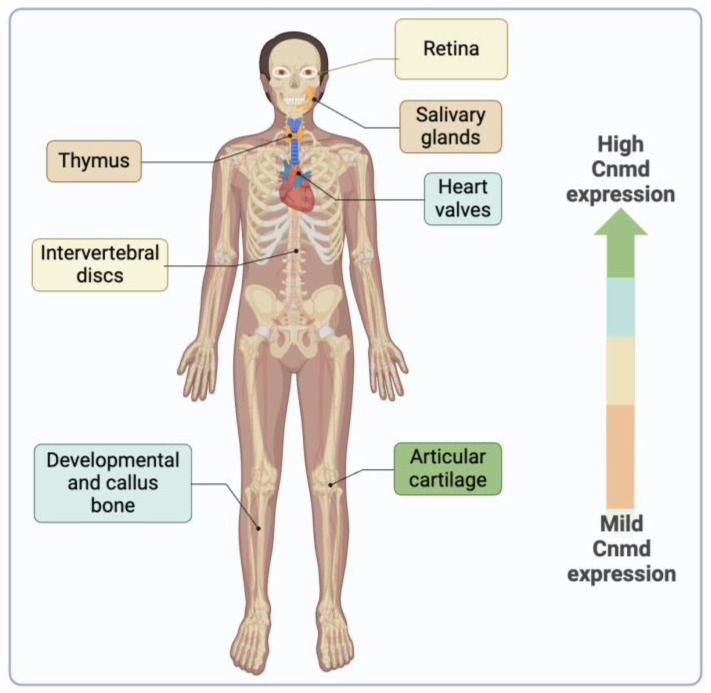
The illustration depicts the presence of Cnmd in diverse tissues beyond its primary location in articular cartilage. The color scale indicates, from greater to milder, the expression of Cnmd.

Cnmd is a protein primarily expressed in healthy adult cartilage and is notably present in the avascular cartilage during endochondral bone formation [6,10,11]. While its predominant expression is observed in cartilage, Cnmd has also been found in various other tissues, such as cardiac valves [68], intervertebral discs [6,69], thymus [70], retina [71,72], and in the decidua during the early implantation period in mice [73] (Figure 6). 

**Figure 7:**
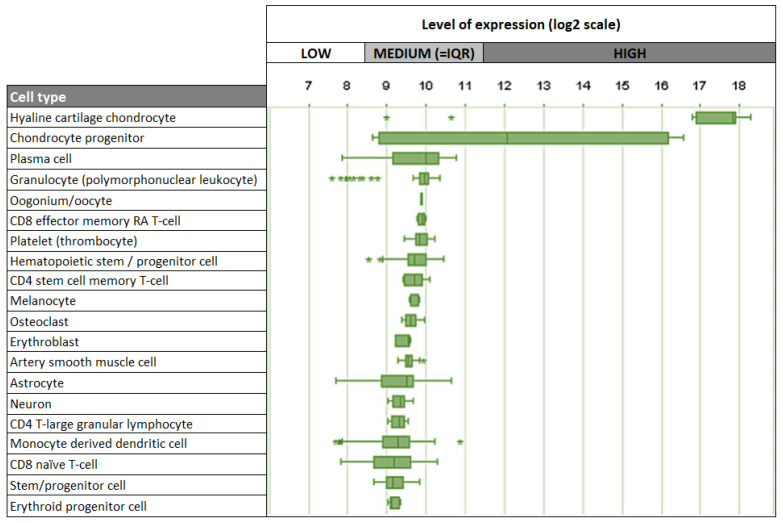
Top 20 cell types expressing Cnmd mRNA in humans. Outliers are represented by stars (*). Adapted from https://genevisible.com/cell-types/HS/Gene%20Symbol/CNMD (accessed on 9 March 2023).

Given its strong association with cartilage, it is not surprising that chondrocytes are the main cell type expressing Cnmd in humans, mice, and rats. Interestingly, besides chondrocytes, other human cell types, including plasma cells, oocytes, melanocytes, and osteoclasts, demonstrate relatively low levels of Cnmd mRNA expression (Figure 7) [74]. This suggests that Cnmd may have additional roles beyond cartilage maintenance and repair in different cell types throughout the body. 

Further research is necessary to validate Cnmd expression at the protein level in vivo and in vitro using different tissues and cell types. Additionally, the effects of different culture conditions, 2D and 3D models, and chemical and biomechanical stimulation on Cnmd down- and up-regulation should be explored. Last but not least, it would also be of interest to clarify the variability in Cnmd expression among different species. Better knowledge of the precise Cnmd expression pattern in various tissues and cell types will provide a more comprehensive concept of its physiological significance.

## 4. Cnmd Functional Characteristics

### 4.1. Cnmd Roles in Cartilage Homeostasis and Callus Fomation during Bone Repair

Ever since its discovery in 1991, several publications have helped to elucidate the different functional roles of Cnmd, which have been mainly related to: (i) homeostatic role in chondrogenesis; (ii) anti-angiogenic properties; and (iii) impact on the biomechanical characteristics of cartilaginous callus during bone repair. A summary of the identified roles of Cnmd is shown in Table 1.

With regard to the Cnmd effect on different cell types, an in vitro study on rabbit growth plate chondrocytes by Inoue et al. (1997) demonstrated that Cnmd stimulates chondrocyte proliferation in the presence of FGF-2 [8]. The anti-angiogenic properties of Cnmd were described for the first time by Hiraki et al. (1997) by purifying Cnmd from bovine epiphyseal cartilage and applying it to bovine carotid artery endothelial cells, which resulted in inhibition of tube formation [10]. In another in vitro study [55], the glycosylated and non-glycosylated recombinant human Cnmd also obliterated the tube morphogenesis of human umbilical vein endothelial cells (HUVEC). Interestingly, the study suggested that the glycosylated form was more potent than the non-glycosylated form.

In two independent studies conducted on mice by Yukata et al. [75,78], a tibial distraction model was created in *Cnmd* KO mice and wild-type (WT) mice. In the first study, static distraction was performed immediately after osteotomy, whereas in the second study, dynamic distraction with a gradual increase over 3 weeks was carried out. In WT mice, *Cnmd* mRNA and protein were localized in the cartilage callus during the reparative process, while the *Cnmd* KO mice exhibited a callus primarily composed of fibrous tissue. This suggested that Cnmd plays a crucial role in proper callus ECM formation during bone repair. In another in vivo study on healthy rats [9], it was found that the full immobilization of the ankle joint led to a significant thinning of articular cartilage but an augmentation of vascular invasion, concomitant with increased expression of HIF-1α and VEGF, in comparison to partial immobilization and free joint movement. The expression of Cnmd in the fully immobilized joint was lower compared to partial immobilization. Thus, it was concluded that Cnmd, together with constant motion, helps to maintain healthy cartilage.

The Cnmd impact on angiogenesis in vivo was revealed when native knee articular cartilage chips from *Cnmd* KO mice showed evident ossification, a process tightly coupled with vascular formation, upon subcutaneous implantation into nude mice [34]. Hayami et al. [79] conducted an experiment in mice to induce human chondrosarcoma using an injection of the OUMS-27 cell line. Once the tumors reached approximately 45 mm^3^ in volume, half of the animals received a subcutaneous administration of human Cnmd, while the other half were given only PBS. The results clearly reported that Cnmd blocked almost 100% of vascular invasion and tumor growth in vivo. In 2014, the 14 kDa form of Cnmd was identified for the first time in mouse hypertrophic cartilage, and it was thought that its cleavage was a mechanism to shut down Cnmd anti-angiogenic capacity, thus permitting vascular sprouting in the calcified zone [5]. 

### 4.2. Cnmd Link to OA

With regards to the Cnmd link to OA, Zhang et al. used a rat model where the meniscus was surgically removed to induce the disease, and lentiviral-based overexpression of Cnmd prevented the formation of hypertrophic chondrocytes [32]. Furthermore, Cnmd application significantly reduced ECM degradation and enhanced the expression of Col2a1 and Acan. The observed delay in OA development suggests that Cnmd could be a promising therapeutic target for managing this degenerative joint disease. In the same study, it was found that the expression of Cnmd was downregulated during OA, and the extent of Cnmd reduction was directly proportional to the severity of the OA condition. This suggests that Cnmd may be involved in the pathogenesis of OA, and its loss contributes to the disturbance of the angiogenic and anti-angiogenic factors. 

The correlation between Cnmd and OA has also been explored in a human model [77]. The localization of CNMD was elucidated within the knee cartilage at different OA stages. Through RT-qPCR, WB, and immunohistochemistry, the expression of CNMD was assessed in parallel to vascular invasion beyond the non-calcified cartilage. The PCR results indicated a decrease in the levels of human *CNMD* mRNA in mild OA compared to young and aged healthy cartilage. Subsequently, there was an abrupt elevation in mRNA levels observed in moderate OA, followed by a return to decreased levels in severe OA. Next, the mature form of CNMD protein was present in the ECM of all regions of healthy cartilage for both young and aged donors, and the CNMD protein levels gradually reduced with OA progression. In line with these results, the immunohistochemistry interestingly showed ECM expression of CNMD in the superficial and middle zones and a slight decrease in the deep zone in the aged healthy group. Across all stages of OA, CNMD protein levels declined in the superficial and middle zones compared to healthy cartilage. Notably, cytoplasmic CNMD expression was elevated in moderate OA. The expression of mature CNMD was inversely correlated with angiogenesis progression in the OA samples. Vascular channels were gradually increased in the deep zone in mild, moderate, and severe OA, which correlates with the progressive loss of CNMD expression. These findings underline the significance of CNMD in OA pathogenesis, and it would be of interest to further investigate different patient cohorts, age groups, gender effects, traumatic OA, and patients suffering osteochondritis dissecans.

Since its discovery, great progress has been made in characterizing Cnmd functions. Nevertheless, new paths need to be undertaken in follow-up studies in order to fully comprehend the Cnmd mode of action. For example, *Cnmd* KO mice can be subjected to various training regimes or to chemical or surgical induction of OA. The possibility of Tnmd compensation should also be clarified. The mechanosensitivity of this gene is still not completely defined, and implementing mechano-stations and bioreactor systems could be of help to progress in this area. How exactly Cnmd can exert opposing effects depending on the cell type remains puzzling, and it will be of great importance upon identification of binding partners to explain the molecular mechanisms. Next, outlining studies that can carefully examine whether Cnmd levels correlate to OA disease stages and hence result in establishing Cnmd as a diagnostic marker, especially if traces are detectable in the synovial fluid, are worthwhile to pursue. As discussed in Section 2, OA is a multifactorial disease in which several catabolic molecules contribute to tissue degradation. Some of these molecules have been screened and their inhibitors/antagonists have been investigated in clinical studies, as potential therapeutics for OA in humans. These include inhibition of IL-1β, FGF, and BMP-7, which mitigates inflammation, the loss of cartilage thickness and volume, and improves the symptomatic state of patients, respectively [52]. While the loss of CNMD may not be the sole factor leading to OA, its dysregulation may result in an imbalance of other molecular players, which collectively may initiate OA pathogenesis. Thus, further research is needed to establish a strong correlation between CNMD and the multifactorial nature of OA. Finally, testing the combination of recombinant Cnmd with cell-based products could pioneer novel therapeutic strategies for quicker and more stable cartilage repair in OA patients.

## 5. Putative Signaling, Pathways, and Related Factors

**Figure 8:**
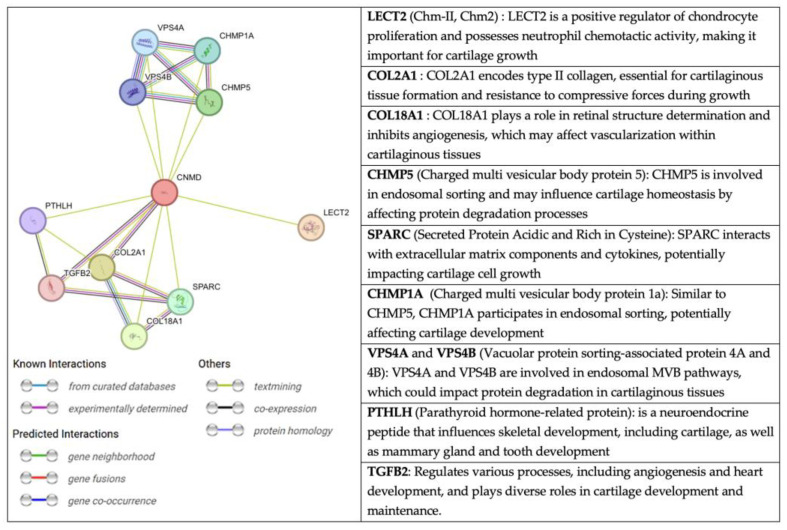
Network of protein-protein interactions with CNMD in humans. The color code of the connectors represents the origin of the interaction between Cnmd (centered red circle) and the related proteins. Extracted from string-db.org (accessed on 11 February 2023).

An interesting perspective, extracted from the String website, on the relationship of CNMD with other molecules is presented in Figure 8. String is a database fed from several sources: automated text mining for various databases, computational predictions of co-expression, genomic context predictions, and interaction results from laboratory experiments. This information is systematically collected and suggests protein-protein associations (putative, physical, and functional) [80,81,82]. Through text mining, CNMD has been found to be co-expressed with COL2A1, COL9A2, COL9A3, and TGF-β2 [12]. The latter are structural elements of cartilage tissue and the vitreous body of the eye.

Another molecule associated with CNMD is chondromodulin II (LECT2, CHM-II). Despite their similar names, CHM-II and CNMD are not homologous genes, and their protein structures greatly differ. CHM-II was purified for the first time from fetal bovine epiphyseal cartilage [83]. The human gene is on chromosome 5q31.1-q32 and encodes a 151 amino acid-long protein. Further cleavage produces the secreted form of CHM-II, which consists of 133 amino acids. This protein is highly expressed in the liver and, to a lesser extent, in bone marrow [84]. Interestingly, a genetic polymorphism in CHM-II (Val58lle) has been associated with rheumatoid arthritis, contributing to the progression of the disease in humans [85]. Moreover, WB analyses of femoral neck cartilage have revealed that CHM-II protein levels have increased in patients with OA. However, the mechanism of CHM-II in OA also remains unclear [86]. Since CHM-II has been linked to OA, it may be of interest to further explore a potential crosstalk with CNMD.

Thus, till today, the CNMD signaling pathway remains unknown; therefore, the following topmost goals should be pursued in future research: (i) the putative network should be validated experimentally; (ii) direct binding partners should be identified at least in the primary sites of protein expression; (iii) upstream and downstream factors of CNMD and their interdependencies should also be outlined and confirmed. Only by accomplishing this level of understanding would it be possible to modulate and steer the CNMD-dependent effect toward the desired outcome. 

## 6. Conclusions and Outlook

Cnmd, identified in 1991, is a tissue-specific matrix component primarily found in healthy cartilage. Predominantly expressed by chondrocytes, Cnmd plays a dual role in chondrocyte proliferation, whilst inhibiting vascular tube formation. Studies have also suggested important functions in callus formation and its quality during bone repair, as well as an intriguing link to OA. Cnmd downregulation in OA correlates with disease severity. Both in vitro and in vivo research has proposed that Cnmd supplementation may delay OA progression, making it a potential therapeutic target. 

Therefore, it is important to continue the research on Cnmd, and the priorities should be as follows: (i) challenging *Cnmd* KO mice with various training regimes or by chemical or surgical induction of OA; (ii) clarifying if cross-compensation between the two homolog genes occurs; (iii) explaining the molecular basis of the dual Cnmd action that goes hand-in-hand with discovering Cnmd binding partners; (iv) establishing Cnmd as a potential diagnostic marker, especially if traces are detectable in synovial fluid; and (v) testing whether the combination of Cnmd with cell-based products could lead to quicker and more stable cartilage repair in OA patients. Taken together, joint multidisciplinary and systematic efforts are required to decipher the enigma behind this gene and thereby unleash its full therapeutic potential.

## Figures and Tables

**Table 1:** Summary of the discovered roles of Cnmd. Publications are given in chronological order.

Reference	Species	Study Type	Main Findings
Yukata et al., 2023 PLOS ONE [75]	Mouse	Role in bone callus formation Role in biomechanical properties In vivo	Mouse model distraction in the tibiae of WT and *Cnmd*^−/−^ mice. The distraction of the tibial bone was lengthened gradually by using an external fixator. A 12-week length of the experiment with eight time points. Eight weeks after the osteotomy, 70% of WT mice showed complete union, compared with 10% of *Cnmd*^−/−^ mice. In the early stages of the experiment, mature Cnmd protein was detected only in the cartilaginous callus of WT mice and co-expressed with Col2a1. The expression of *Cnmd* mRNA in WT mice was upregulated during the early stages (weeks 1–3) of mechanical stress. The formation of cartilage callus during distraction osteogenesis was suppressed in *Cnmd*-deficient mice. The subsequent bone formation and remodeling were delayed and partially failed in *Cnmd*^−/−^ mice, where delayed and incomplete fibrotic callus was observed. This implies that Cnmd plays a role in the production of Col2 and other typical compounds of callus cartilage. Cnmd is necessary for cartilage callus formation, and its deficiency leads to delayed bone consolidation and remodeling after fracture.
Di Pauli von Treuheim et al., 2021 Cartilage [69]	Mouse caudal and lumbar vertebrae	Role in biomechanical properties In vivo	Caudal spines were harvested from skeletally mature 9-month-old WT, *Tnmd* KO, and *Cnmd/Tnmd* KO mice. The caudal discs underwent mechanical assessment: axial tension/compression, creep, and torsion to failure. Several biomechanical properties, such as axial compressive stiffness and torsional failure strength, were worsened by the absence of *Cnmd/Tnmd* in comparison to WT and *Tnmd* KO only, suggesting a sort of compensatory role for Cnmd in the single mutation of *Tnmd.* Lumbar discs were histomorphologically scored using an intervertebral disc (IVD) scoring system [76] that evaluates the structure, cell infiltration, mineralization extent, and evidence of fissures. The *Cnmd/Tnmd* KO group showed a greater degeneration severity of lumbar intervertebral discs characterized by fissures and loss of lamellar structure in the annulus fibrosus and larger nucleus pulposus penetration. The lack of *Cnmd* and *Tnmd* genes severely degrades the morphology of IVD. The overall biomechanical performance of the organ was significantly reduced when Cnmd and Tnmd were deficient. The expression of both genes can prevent IVD degeneration.
Deng et al., 2017 Molecular Medicine Reports [77]	Human	Role in cartilage homeostasis Role in anti-angiogenesis Ex vivo	Articular cartilage from the knee and femoral head was obtained from young and aged donors with conditions varying from healthy to different stages of OA. CNMD expression in the non-calcified zone was evaluated from the collected tissue via qRT-PCR, immunohistochemistry, and WB. The presence of cytoplasmic CNMD was unaltered between chondrocytes of young and aged healthy donors, whereas the expression of Cnmd in the ECM was present in the superficial and middle zones, with a small decrease in the deep zone for the aged group. In all stages of OA, Cnmd protein levels decreased in the superficial and middle zones of the ECM, compared to healthy cartilage. Interestingly, the cytoplasmic expression of CNMD was higher in moderate OA relative to mild OA. In severe OA, CNMD was predominantly trapped in the ECM of the middle zone, surrounding the clusters of chondrocytes. The CNMD expression in the ECM was correlated with angiogenesis progression in the OA samples. Vascular channels were gradually increased in the deep zone in mild, moderate, and severe OA, which correlates with the progressive loss of CNMD expression. These results suggest that Cnmd expression helps healthy articular cartilage resist vascular invasion.
Zhang et al., 2016 Osteoarthritis and Cartilage [32]	Human Rat	Role in cartilage homeostasis Role in anti-angiogenesis In vitro and ex vivo	In the animal model, OA was surgically induced by removing the meniscus. OA was surgically induced in rats by removing the meniscus. ○ At different time points after surgery, animals received an intra-articular injection of lentivirus containing *Cnmd* cDNA plasmid (LV-Cnmd). ○ Cnmd, Col2a1, and ACAN expression increased in OA rats by the LV-Cnmd injection. ○ Reduction of proteoglycan depletion in OA rats treated with LV-Cnmd. ○ The overexpression of Cnmd resulted in a decrease in catabolic markers such as COL10a1, MMP-13, and VEGFA. mRNA was isolated from human and rat OA cartilage. Human and rat OA cartilage was used for qRT-PCR, WB, and immunohistochemical staining with VEGFA, Cnmd, ACAN, HIF-2α, MMP-13, and other anti-angiogenic factors such as tissue inhibitor metalloproteinase-1 (TIMP-1) and TIMP-2. ○ VEGFA and Cnmd were expressed in mild and severe OA. ○ In mild OA cartilage, Cnmd was strongly present in the cytoplasm and surrounding ECM, whereas VEGFA was detected in chondrocytes. ○ In severe OA, Cnmd decreased in comparison to mild OA, and VEGFA expression was stronger. ○ qRT-PCR and WB confirmed the downregulation of Cnmd, TIMP-1, and TIMP-2, while VEGFA was upregulated. ○ Human angiogenesis PCR results showed an increased expression of angiogenic cytokines and the downregulation of most of the anti-angiogenic cytokines in OA samples. Articular cartilage chondrocytes (ACCs) were re-differentiated in pellet form and grown in differentiation media containing TGF-β3. The expression of chondrogenic, angiogenic, and anti-angiogenic markers was evaluated via Cnmd, VEGFA, and Col2a1 immunohistochemical staining. ○ Cnmd and Col2a1 levels gradually increased along the re-differentiation process, opposite to VEGFA expression. ○ Cnmd expression levels were higher in the 3-week re-differentiated ACCs compared to 1-week. ○ Gene expression of Cnmd and Col2a1 gradually increased, while VEGFA decreased. ACCs were also transfected with Cnmd lentivirus or siRNA and subsequently stimulated with TNF-α to promote hypertrophy. Cnmd and HIF-2 pathway interactions were investigated. ○ After 48 h, cells were collected for qRT-PCR and Western blotting (WB) analyses. ○ Cnmd showed to prevent the expression of TNF-α-induced molecules such as Col10a1, MMP-13, and VEGFA. ○ Results suggest that the presence of Cnmd has an influence on preventing HIF-2α activity. The expression of Cnmd could prevent hypertrophy in chondrocytes, decreasing the progression of matrix degradation and enhancing the expression of Col2a1 and ACAN. The progression of OA decreased the expression of Cnmd and initiated the expression of VEGFA and other catabolic genes.
Zhu et al., 2015 Tissue Engineering Part A [34]	Mouse	Role in cartilage homeostasis In vivo	Articular cartilage was collected from 3-day-old and 9-week-old WT and *Cnmd*^−/−^ mice. Chondrocytes were harvested to evaluate phenotypes. ○ Morphological analysis and histological staining did not show differences in cell shape or glycosaminoglycan (GAG) content. ○ COL-II, COL-X, and VEGFA immunostaining showed no differences in expression between phenotypes. Ectopic subcutaneous implantation of articular cartilage from *Cnmd*^−/−^ and WT into nude mice. ○ After 16 days of implantation, evident ossification of Cnmd^−/−^ cartilage. ○ At the same timepoint, WT cartilage maintained its healthy cartilaginous characteristics. ○ GAG and COL-II expression were found only in the ectopic WT cartilage. ○ VEGFA expression was detected only in the ectopic Cnmd^−/−^ cartilage implant. This suggests that Cnmd does not influence articular cartilage development but is important for the preservation of cartilage homeostasis and the prevention of cartilage ossification.
Miura et al., 2014 PLOS ONE [5]	Mouse C57BL/6 Rat Wistar/ST Human umbilical vein endothelial cells (HUVECS)	Role in anti-angiogenesis In vivo and in vitro	Proteins were extracted from 3-week-old rat costal cartilage. A new form of Cnmd was identified by immunoblotting. It is 14 kDa generated by the cleavage of the N-terminal at Asp37–Asp38, in comparison to the intact 25 kDa glycoprotein. HUVECS were incubated with human recombinant 14 kDa and 25 kDa Cnmd proteins, and HUVEC migration was assessed by a modified Boyden chamber assay. The 14 kDa Cnmd showed a poor inhibitory effect on the VEGF-A-induced migration of the HUVECs compared to the 25 kDa form. Hindlimbs from mouse embryos were collected to perform immunostaining against the 25 kDa and 14 kDa versions of Cnmd. The ECM in the hypertrophic and calcified zones of articular cartilage lacks intact 25 kDa Cnmd but instead contains only the truncated 14 kDa Cnmd. Although 25 kDa and 14 kDa Cnmd contain the C-terminal and disulphide bridges that are critical for the anti-angiogenic activity, the short form of Cnmd is the only one found in hypertrophic cartilage, and it was shown to have a very little inhibitory effect on the VEGFA-induced migration of vascular endothelial cells.
Kondo et al., 2011Bone and Mineral Metabolism [55]	HUVEC Rat chondrocytes	Role in anti-angiogenesis In vitro	To compare the effects to the natural glycosylated form, a non-glycosylated recombinant human Cnmd (NG-hCnmd) was produced by cloning mature human Cnmd into the NcoI and BamHI sites of the pET-11a plasmid vector and expressing it in *E. coli.* Rat growth plate chondrocytes as well as HUVECS were treated with different concentrations of glycosylated and NG-hCnmd. After 22 h of incubation, NG-hCnmd and glycosylated Cnmd stimulated the synthesis of DNA in the presence of recombinant human FGF2 in a dose-dependent manner. Similarly, colony formation in agarose was stimulated in chondrocytes treated with NG-hCnmd and glycosylated Cnmd. It was shown that both types of Cnmd inhibit the tube morphogenesis of HUVECS in vitro. However, to obtain similar results, the non-glycosylated Cnmd required a concentration ten times higher than the glycosylated protein in the colony formation assay as well as in the tube morphogenesis assay. Cnmd is a glycoprotein that has an inhibitory effect on angiogenesis and demonstrates both growth factor properties in chondrocytes and inhibitory effects in vascular endothelial cells. The glycosylation of the N-terminal domain is essential for maintaining structural Cnmd integrity and carrying out its biological function.
Miura et al., 2010Experimental Cell Research [67]	HUVEC Mouse BALB/c	Role in anti-angiogenesis In vivo and in vitro	Poly-2-hydroxyethyl methacrylate (HEME) pellets containing commercially available VEGF-A (VEGF_165_) (Sigma) and recombinant human Cnmd corresponding to the region Glu^215^-Val^334^ of the human ChM-I precursor were implanted into the corneas of mice. Fourteen days after implantation, the corneas were collected and immunostained with CD31. The corneas treated with VEGF-A and Cnmd showed effective suppression of new blood and lymphatic vessels compared to only VEGF-A application. HUVECs were treated with and without recombinant human Cnmd and then VEGF-A-stimulated. The inhibition of the VEGF-A-induced migration was directly proportional to the Cnmd dosage. Immunofluorescence in the actin filaments of HUVECs co-treated with Cnmd and VEGF-A showed a disrupted cytoskeleton with poor organization of the remaining actin filaments. Upon Cnmd treatment, VEGF-A-stimulated HUVECs did not display lamellipodia at the leading edge, typical of a migrating cell. The presence of human recombinant Cnmd in cell culture suppressed VEGF-A-stimulated migration of endothelial cells. The authors suggest that Cnmd disturbs the reorganization of actin filaments in HUVECs, delaying the lamellipodial extensions and thus reducing the motility of treated cells.
Sakamoto et al., 2009 Connective Tissue Research [9]	Wistar Rat	Role in cartilage homeostasis Role in anti-angiogenesis In vivo	Twelve-week-old rats were divided into three groups: control, full ankle joint immobilization in full plantar flexion, and ankle joint immobilization with continuous passive motion (CPM). In the third group, the fixation was temporarily removed to apply CPM. After 4 weeks, ankle joints were analyzed. The immobilized and CPM showed significant thinning of the articular cartilage. In the full-immobilized-joint group, it was noticed that there was an increased expression of HIF-1α and VEGF and low levels of Cnmd with respect to the control. Cnmd expression was decreased in the immobilized mice, whereas no significant difference was found between the control and CPM groups. The number of vascular vessels was significantly higher in immobilized mice compared to the other groups. The presence of Cnmd, together with exercise, helps with the maintenance of healthy cartilage. In comparison, immobilization of the joints promoted vascular invasion and cartilage thinning, which was paralleled by low Cnmd expression. This decreased expression of Cnmd may contribute to the formation of numerous vascular channels invading the calcified zone of the subchondral bone.
Yukata et al., 2008 Bone [78]	Mouse	Role in bone callus formation In vivo	WT and Cnmd^−/−^ mice that were 9- or 10-week-old were examined for distraction in the right tibiae. An osteotomy was surgically performed, and an external fixator was used to stabilize the bone fracture. Radiographs were taken on days 7, 14, and 21 to evaluate the microstructure in the callus. ○ The formation of internal and external calluses was noted in the two groups. ○ At day 14, the intramedullary calcified area within the internal callus was larger in Cnmd^−/−^ compared to WT. ○ The bone marrow space was smaller in the mutant group during the observation period. Toluidine blue staining was carried out to analyze the cartilaginous area during the healing process. ○ Callus formation was observed from day 5 in the periosteal region of WT, while in Cnmd^−/−^, the metachromasia of the toluidine blue staining appeared from day 7 only at the fracture site. ○ A bony callus was found in the *Cnmd*^−/−^ mice instead of the cartilage callus. Immunohistochemistry was performed to examine the location of Cnmd in the right tibiae and surrounding tissues. ○ The expression zones were in line with the results given by the toluidine blue staining. ○ Cnmd signal faded with the progression of endochondral ossification, and it was found only in immature chondrocytes but not in hypertrophic mature chondrocytes. RNA from the soft tissue and bone marrow was extracted at different time points. Real-time PCR was performed for *Cnmd*, *Sox9*, *Col2a1*, and *Col10a1*. ○ The expression of Cnmd mRNA in WT mice was markedly upregulated during fracture repair. ○ In Cnmd-deficient mice, the levels of *Col2a1* and *Sox9* were upregulated. This suggests that the absence of Cnmd affects the proliferation of chondrocytes and their subsequent hypertrophic maturation in the external callus. While the direct effect of Cnmd on bone remains vague, it was shown that the absence of Cnmd delays the union of a fractured bone.
Yoshioka et al., 2006 Nature Medicine [68]	ICR Mouse Wistar Rat Human	Role in anti-angiogenesis In vivo and In vitro	Cardiac valves of 2-month-old and naturally aged 20-month-old Cnmd KO mice and WT mice as controls were collected to investigate the molecular mechanisms involved in the avascularity of cardiac valves. The histological images showed that cardiac valves from young *Cnmd* KO mice were enlarged compared to young WT. ○ The expression of *Cnmd* mRNA appears at day 9 of embryonic development and is maintained during adulthood. ○ The thickness of valvular walls in *Cnmd* KO was larger compared to controls. These results were also suggested by echocardiography results. ○ The cardiac valves of *Cnmd* KO presented new capillary-like structures as well as calcium deposits, identified via von Kossa staining. WB identified the expression of Cnmd in the cardiac valves of rats and healthy human tissue. Specimens of healthy and pathologic human cardiac valves were collected for histological staining and immunostaining. ○ Healthy tissue did not present blood vessels, and the expression of Cnmd was found in the laminae fibrosa, spongiosa, and ventricularis layers. ○ In pathologic samples, several blood vessels were present, and the downregulation of Cnmd was evident in these regions. Vascular interstitial cells (VICs) from cardiac valves were isolated from 5-week-old rats and cultured to investigate whether the cells produce Cnmd. VICs were also treated with *Cnmd* siRNA to study the effects. ○ Immunohistological results showed the presence of Cnmd within the cytoplasmic region of VICs and the ECM. Such expression did not overlap with VEGF-A signals. ○ Cnmd was also present in the media. ○ Cnmd expression was silenced by treating VICs with *Cnmd* siRNA. Human coronary artery endothelial cells (HCAECs) were cultured in normal media and with cell media harvested from VICs expressing Cnmd and from VICs treated with siRNA. Imaging analysis was carried out after 6 h of culture on Matrigel. ○ HCAECs in normal media generate capillary-like structures. ○ In contrast, the cells in media containing Cnmd produced 75.9% less prominent tubular structures compared to the control. ○ HCAECs cultured with siRNA-treated VIC media recovered 62.9% of their capacity to form such structures compared to the control. HCAECs were cocultured with VICs-expressing Cnmd and VICs-siRNA-treated. ○ Staining with Annexin V and Propidium Iodide showed morphological changes, suggesting apoptosis. ○ Inhibition of migration in HCAECs was verified through a Boyden chamber assay. Loss of *Cnmd* leads to neovascularization and calcification in the cartilage-like matrix of cardiac valves. These observations underscore the critical role played by Cnmd in preserving normal valvular function and impeding angiogenic processes that could contribute to heart pathological conditions.
Hayami et al., 1999 Federation of European Biochemical Societies [79]	BALB/c Nude Mouse	Role in anti-angiogenesis In vivo	Four-week-old mice were used to induce chondrosarcoma through a subcutaneous injection in the back of a human chondrosarcoma cell line (OUMS-27). Recombinant human full-length Cnmd was expressed in Chinese hamster ovary (CHO) cells and purified. When the tumors reached a volume of around 45 mm^3^, half of the population received a subcutaneous administration of human recombinant Cnmd diluted in PBS, whereas the other half received PBS only. Results showed that Cnmd blocked almost entirely the vascular invasion and tumor growth. Cnmd can contribute to the resistance of cartilage against angiogenesis during malignant transformation, implying its potential as a therapeutic candidate for solid tumors.
Inoue et al., 1997 Biochemical and Biophysical Research Communications [8]	Bovine Rabbit	Role in cartilage homeostasis In vitro	Chondrocytes from the ribs of rabbits were cultured and stimulated with FGF-2 and different concentrations of recombinant human Cnmd. Colony formation of chondrocytes in agarose was potentiated with the treatment of FGF-2 and Cnmd, whereas the colony formation was weaker in the presence of Cnmd alone. Higher doses of Cnmd (>200 ng/mL) were required for chondrocytes to form colonies. Cnmd stimulation maintained the cellular morphology of chondrocytes. The synthesis of proteoglycans was stimulated by the presence of Cnmd in a dose-dependent fashion. In the presence of FGF-2, Cnmd might participate in a signaling pathway to encourage chondrocyte renewal.
Hiraki et al., 1997 Journal of Biological Chemistry [10]	Bovine carotid artery endothelial cells (BCAE) and developing bovine tails	Role in anti-angiogenesis In vitro	BCAE cells were used to study the formation of tube-like networks. After 7 days of cell culture treated with recombinant bovine Cnmd, there was no trace of tube formation. In situ hybridization and immunohistochemical studies for Cnmd were performed in developing bovine bone. Results showed Cnmd expression in the avascular cartilage region of developing bone, but no expression was found in calcifying cartilage. This study showed for the first time the regulatory role of Cnmd in vascular invasion during endochondral bone formation.
